# Lack of hemochromatosis in Saint Augustine in his studio by Sandro Botticelli

**DOI:** 10.1007/s40618-024-02367-6

**Published:** 2024-04-27

**Authors:** N. Kluger

**Affiliations:** 1 Department of Dermatology, Allergology and Venereology, Helsinki University Hospital & University of Helsinki, Helsinki, Finland; 2 Société Française des Sciences Humaines sur la Peau (SFSHP), Maison de la Dermatologie, 10, Cité Malesherbes, 75009 Paris, France

**Keywords:** Art, Hemochromatosis, Iconodiagnosis, Painting

**Fig. 1:**
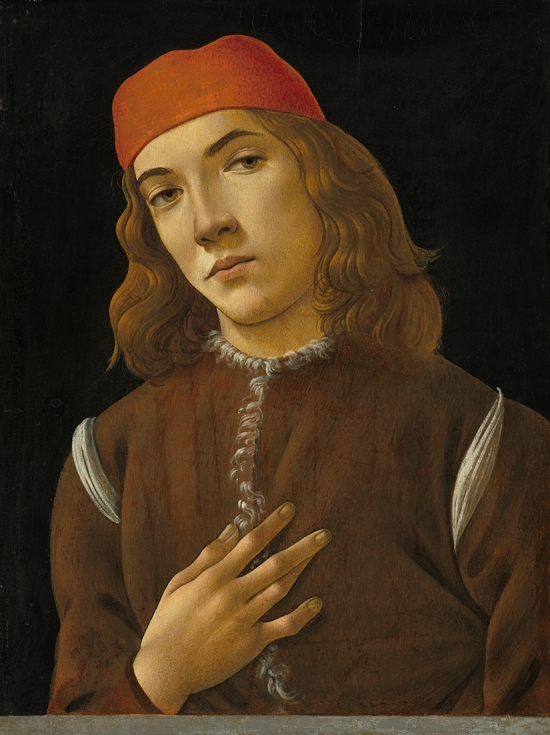
Sandro Botticelli (1446–1510) *Portrait of a Youth,* c. 1482/1485 (*Courtesy National Gallery of Art, Washington*). The hands and nails display the same features as *Saint Augustine in his studio* in the Church of Ognissanti

Dear Editor,

I read the recent letter by de Campos that believed to have identified features of hemochromatosis in the fresco painting of Saint Augustine (1480) by Sandro Botticelli in the Church of Ognissanti in Florence [1]. The author based his interpretation solely on the "physical" examination of Saint Augustine outside of any contextualization of the fresco. The author interpreted Saint Augustine’s skin tone as a sign of melanoderma. Saint Augustine or Augustine of Hippo (354–430 AD) was a North African of Berber origin and born in Thagaste, a town located in the far east of what is now Algeria. Therefore, Augustine would be expected to display a darker skin complexion than Europeans. Besides, as the fresco is more than 500 years old, it has inevitably suffered the ravages of time as visible in the figure in the article [1]. Original colors may have faded or darkened with time. Analyzing the colors nowadays *as if* they were the same at the time of Botticelli is an anachronistic mistake. Only a proper restoration would bring the true original colors back. The author states that Saint Augustine has whitened nails, suggesting Terry nails or total leukonychia, a known sign of liver cirrhosis. A careful examination shows that the nails display a white linear streak on the radial side on the right hand and on the distal lateral part on the left one. Furthermore, the forehead, tip of the nose and dorsum of the right-hand of the saint display similar whitening. These parts of the body seem to be illuminated by a source of light coming from the upper left part of the fresco, towards which the saint’s gaze is directed. The shadows on the clothes and the tablecloth also indicate a source of light from the left side. De Campos sees arthritis on the fingers of Saint Augustine. It is not the first time that Botticelli is suspected erroneously to have depicted arthritis [2]. When it comes to analyzing hands or posture, it is important to rule out a stylistic habit of the artist [2]. In another artwork by Botticelli, the *portrait of Youth* (c. 1482/1485 National Gallery of Art, Washington), the sitter displays similar finger shapes, and even similar whitening of the nails due to light exposure (Fig. 1). Lastly, the author considers that the dramatic gesture of Saint Augustine is a "discernible sign of malaise and fatigue" [1]. The historical and artistic aspects of the fresco need to be taken into account [3]. Botticelli’s fresco is closely related to Ghirlandaio’s *Saint Jerome in his study,* painted the same year and facing the fresco in the church [3]. Besides, various interpretations have been given to Botticelli’s fresco. Some see a paradigmatic image of the ideal scholar or sage or the icon of a saint. A more recent interpretation is that Botticelli represented an apocryphal event in Augustine’s life [3]: the miraculous visitation of Jerome at the very same moment he died in Jerusalem. When writing a letter to Jerome, Augustine experienced an indescribable light entering his cell with unknown odors and fragrance [3]. The pose of Saint Augustine, his gaze and the whitening of his skin and nails are in line with this interpretation.

The practice of iconodiagnosis must respect a certain number of criteria when it comes to publishing a case [4]. The observer cannot simply look at an artwork and interpret it without knowledge of the historical, artistic, cultural, or social context of the period, sitter's patho-biographical information, the style of the artist, or the art history surrounding the work. Overlooking such additional work may lead to erroneous diagnosis. It is very unlikely that Botticelli represented any sign of hemochromatosis.
